# Ultrastructural, Chemical, and Wettability Insights into the Interaction between Novel Natural Dentifrices and Nanohybrid Composites: A Multi-Technique Microscopy Approach

**DOI:** 10.1021/acsomega.6c09534

**Published:** 2026-10-24

**Authors:** Giovanna dos Santos S. Teixeira, Ilene Cristine R. Cesar, Luís Eduardo S. Soares

**Affiliations:** Laboratory of Dentistry and Applied Materials, Research and Development Institute, 67655 University of Vale Do Paraíba, Sao Jose dos Campos 12244-000, Brazil

## Abstract

This study aims to provide a multimodal ultrastructural and microanalytical characterization of the effects of essential-oil and ozonated-oil toothpastes on a zirconia-reinforced nanohybrid composite resin. Fifty composite specimens were distributed into five groups: artificial saliva (AS), conventional toothpaste Colgate Total 12 (CT), Colgate Natural Extracts Reinforced Defense gel (CN), DoTERRA on guard (DT), and Ozonecare gel (OZ). Following simulated brushing, a comprehensive surface assessment was performed using micro energy-dispersive X-ray fluorescence spectrometry (μ-EDXRF) for elemental mapping, contact angle (CA) measurements for surface-free-energy analysis, and scanning electron microscopy (SEM) for topographical evolution. Dentifrice components were further characterized by SEM, μ-EDXRF, and FT-Raman spectroscopy. A significant decrease in oxygen was detected only in the CN group. Increased Si, Ba, and Ti content was observed in the AS and CN groups. Reductions in CA occurred after brushing with CT and DT. Increased CA was found in AS. No significant changes in wettability were found using CN or OZ. SEM analysis revealed extensive morphological impact and increased filler exposure in samples treated with CT, moderate degradation with DT, and minimal morphological impact with CN and OZ. Particle analysis demonstrated the presence of larger, irregular abrasives in CT, silicate-rich particles in DT, and homogeneous fine particles in both CN and OZ. FT-Raman spectroscopy detected TiO_2_ exclusively in CT and prominent silicate bands in DT. The toothpaste formulation significantly affected the composition, wettability, and morphology of the composite resin. Notably, OZ gel preserved both the elemental composition and surface morphology of the composite.

## Graphical abstract

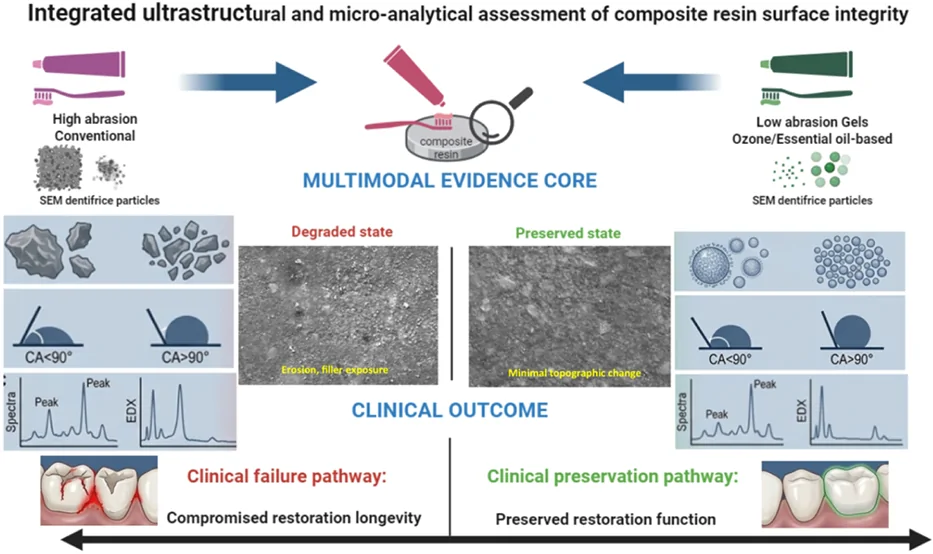

## 1 Introduction

The growing societal emphasis on aesthetics has driven significant advancements in the dental esthetic materials industry. Each year, new materials and techniques are introduced into the market.^1^

Composite resins are utilized for both small, simple restorations and direct veneers. Because of their continuous exposure to food and saliva, maintaining optimal oral hygiene is essential to prevent plaque accumulation. Regular tooth brushing is necessary to remove plaque from both natural teeth and restorative materials.^2,3^

Toothpaste contains abrasive materials that help remove plaque, tartar, and stains from the surfaces of teeth. When these abrasives come into contact with teeth or restorative dental materials, they create friction as the two surfaces move against each other. If the abrasive is rougher, then it can create grooves or cuts in the smoother surface, which is known as abrasive wear.^4^ This wear can result in a rough texture on restorative materials,^5^ negatively affecting oral hygiene. Rough surfaces are more likely to adhere plaque and food particles, making it harder to maintain clean teeth.^2,4,6^

Brushing with toothpaste can lead to abrasion on the surface of the composite resin. During this abrasion process, the abrasive agents in the toothpaste scratch the matrix of the composite resin, causing erosion on the surface of the restorative material.^2,7,8^ Toothbrush abrasion also is the most common factor that influences surface properties and color stability of composites in vivo.^9^ Differences in toothpaste abrasivity are also related to product consistency, where gel toothpastes contain silica as their abrasive agent and are considered less abrasive.^10^

Another factor that influences the properties of the composite resin during brushing is the phenomenon of sorption and solubility.^11^ The occurrence of those phenomena serves as precursors to chemical and physical processes, such as oxidation and hydrolysis, swelling, plasticization, and softening.^12^ As a consequence, the properties of composite resin are modified, thus creating biological concerns about the material biocompatibility.

In recent decades, there has been an increasing interest in environmentally sustainable, natural, and eco-friendly products.^13^ This trend has prompted the personal care and hygiene industry to invest in research and development of products made with natural ingredients and extracts. Additionally, the appeal of products that are free from chemical additives and harmful side effects, coupled with the biological advantages of natural extracts, has fueled growing demand for products with natural formulations.^14^

In recent years, there has been increasing interest in biocompatible products, which has resulted in a rise in the number of oral hygiene products containing plant extracts on the market. This change is driven by the demand for ingredients that are more effective against plaque bacteria and gingivitis.^15^

Essential oils have gained significance in dentistry alongside ozone, particularly as components of toothpaste.^13^ These substances are included in toothpaste because of their antimicrobial, anti-inflammatory, and analgesic properties. When ozone is incorporated into toothpaste, it can help maintain oral hygiene around restorations, thereby reducing the risk of secondary caries and gum inflammation.^16^

Previous studies have evaluated the effects of ozonated olive oil-containing dentifrice on enamel color change and microhardness, demonstrating a significant improvement in color without compromising microhardness, as well as dentin tubule occlusion as a promising approach for treating dentin hypersensitivity.^17^ However, investigations into the specific interaction of toothpaste containing essential oils or ozonated oils with restorative materials remain poorly understood. Given the rapid proliferation of new oral hygiene products and the potential risk they pose to the clinical longevity of composite restorations, a systematic investigation is required. Therefore, this study aims to provide an integrated microanalytical and morphological assessment of a zirconia-reinforced nanohybrid composite resin subjected to simulated brushing with essential oil and ozonated oil formulations. The investigation focuses on elucidating the complex interplay between dentifrice chemistry and the material’s surface integrity, specifically mapping alterations in elemental stoichiometry, topographic evolution, and physicochemical stability.

## 2 Materials and Methods

Specimens were randomly assigned (n = 10 per group) to five experimental conditions (Table 1):

AS – samples brushed only with artificial saliva (negative control).
CT – conventional toothpaste (Colgate Total 12 Clean Mint, positive control)
DT – essential-oil toothpaste without fluoride (DoTERRA On Guard)
CN – essential oil toothpaste with fluoride (Colgate Natural Extracts Reinforced Defense)
OZ – ozonated olive-oil toothpaste (OzoneCare)

**Table 1:** Description of Experimental Groups

Group	Material Trade Name And Description	Basic composition (manufacturer data)	Fluoride
AS	Artificial saliva, negative control (NostraFórmula; Brazil)	1.5 mmol L^–1^ Ca(NO_3_)_2_ H_2_O; 0.9 mmol L^–1^ Na_2_HPO_4_ 2H_2_O; 150 mmol L^–1^ KCl; 0.1 mol L^–1^ H_2_NC(CH_2_OH)_3_ (TRIS); 0.05 mg mL^–1^ NaF.	-
CT	Colgate Total 12 Clean Mint, positive control (Colgate-Palmolive; Brazil)	Glycerin, Water, Hydrated Silica, Sodium Lauryl Sulfate, Arginine, Flavor, Cellulose Gum, Zinc Oxide, Poloxamer 407, Tetrasodium Pyrophosphate, Zinc Citrate, Benzyl Alcohol, Xanthan Gum, Cocamidopropyl Betaine, Sodium Fluoride, Sodium Saccharin, Phosphoric Acid, Sucralose, Titanium Dioxide (CI 77891), Pigment Blue 15 (CI 74160), Pigment Green 7 (CI 74260), Eugenol. Contains Sodium Fluoride (1450 ppm Fluoride). Active Ingredients: Sodium Fluoride, Zinc Citrate, Zinc Oxide.	Sodium fluoride (1450 ppm fluoride)
CN	Colgate Natural Extracts Reinforced Defense (Colgate-Palmolive; Brazil)	Sorbitol, Water, Hydrated Silica, Sodium Lauryl Sulfate, Flavor, Peg-12, Cellulose Gum, Tetrasodium Pyrophosphate, Cocamidopropyl Betaine, Sodium Saccharin, Sodium Fluoride, Mica, Aloe Barbadensis Extract, Titanium Dioxide (Ci 77891), Ci 47005, Ci 74260, Benzyl Alcohol, Dipentene, Citral, Eugenol.	Sodium fluoride (1100 ppm fluoride)
DT	doTERRA on guard (doTERRA, EUA)	Glycerin, Water (Aqua), Hydrated Silica, Calcium Carbonate, Xylitol, Hydroxyapatite, Cellulose Gum, *Mentha piperita* Oil, Xanthan Gum, Stevia Rebaudiana Leaf Extract, Sodium Lauroyl Sarcosinate Carmellose Sodium, Citrus Aurantiumdulcis Peel Oil, *Cinnamomum zeylanicum* Bak Oil, *Eugenia caryophyllus* Bud Oil, *Cinnamomum zeylanicum* Leaf Oil, *Gaultheria procumbens* Leaf Oil, *Eucalyptus globulus* Leaf Oil, Commiphora Myrha Oil, *Rosmarinus officinalis* Leaf/Stem Oil, Sodium Chloride, Sodium Glycolate.	-
OZ	Ozoncare dental gel (Philozon, Brazil)	Glycerin, water, hydrated silica, sodium lauroyl sarcosinate, xanthan gum, ozonated olive oil, xylitol, *Mentha piperita* oil, sodium benzoate, benzyl alcohol, *Calendula officinalis* flower extract, *Eucalyptus globulus* leaf oil, menthol, *Citrus aurantifolia* oil, *Citrus grandis* peel oil, *Melaleuca alternifolia* leaf oil.	-

The sample size (n = 10) was determined based on standardized and widely established protocols in the literature for *in vitro*-simulated tooth-brushing studies.^18,19^

### 2.1 Composite Resin Sample Preparation

Composite samples were obtained using a nanohybrid composite resin (FORMA, Ultradent – BW shade). The FORMA composite resin contains bis-GMA, TEGDMA, Bis-EMA, and UDMA. Its formula contains a zirconia/silica inorganic filler combination and barium glass. The resin composite was compacted in two 2 mm-thick layers into the Teflon mold (7 mm diameter × 2.5 mm deep). Then, a Mylar strip was placed on top of each resin specimen, and light polymerization was performed with a light-emitting diode source for 20 s (VALO Grand; ULTRADENT, South Jordan, UT, USA; 385–515 nm; light output: 1000–3200 mW/cm^2^).

The specimens were then stored in a lightproof container. Each recipient was named and numbered to allow data comparison before and after the treatments.^1,19^

### 2.2 Initial μ-EDXRF Analysis

Before brushing, the baseline composition of each composite resin specimen was measured by micro-energy-dispersive X-ray fluorescence spectrometry (μ-EDXRF). The samples were analyzed directly on the equipment (μ-EDXRF; model μ-EDX-1300, Shimadzu, Kyoto, Japan), without the need for prior sample preparation, such as fixation or metallization process.

This initial analysis was used as a standard reference point for the subsequent evaluation of any potential changes resulting from the treatments performed (first analysis, n = 50) (Figure 1).

**Figure 1:**
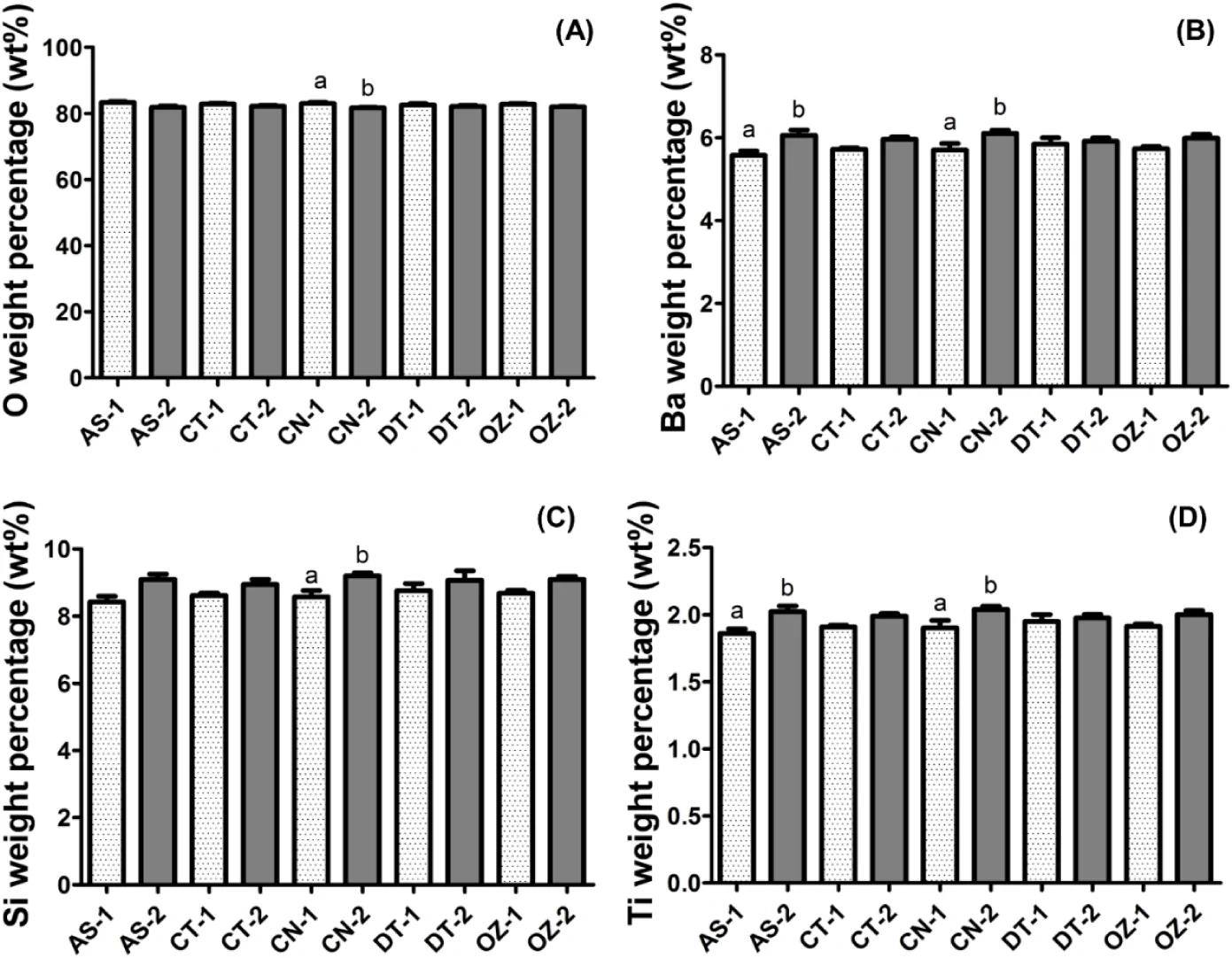
Mean and standard deviations of the O (A), Ba (B), Si (C), and Ti (D) weight percentages in the composite resin obtained by μ-EDXRF before and after simulated brushing in the experimental groups. Statistical comparisons: different letters indicate statistically significant differences between the initial and final measurements after brushing. Legend: AS—artificial saliva, CT—Colgate Total 12 Clean Mint Control, DT—DoTERRA On Guard, CN -Colgate Natural Extracts Reinforced Defense Toothpaste, and OZ—OzoneCare Toothpaste.

The voltage in the tube was set at 40 kV, with automatic adjustment of the current. Three spectra from each specimen were collected before treatment. The measurements were performed with a count rate of 100 s per point. Element oxygen (O) was used as a chemical balance. Energy calibration was performed by using equipment-specific internal standards.

### 2.3 Surface Wettability: Contact Angle Characterization

The contact angle measurements were used to assess the wettability of the untreated composite resin (first analysis, n = 50) using a Drop Shape Analyzer system (DSA 100, Top View Analyzer TVA 100, Krüss) (Figure 1). Each sample was affixed to a mobile platform, and a 3-μL water drop was dispensed onto the surface of the sample. The system recorded the kinetics of the drop shape change, with three measurements taken for each sample.

### 2.4 Simulated Brushing

The brushing simulation was conducted using the selected toothpaste for each group. A paste slurry was prepared by mixing the commercial toothpaste with artificial saliva (“Nostrafórmula” Compounding Pharmacy, São José dos Campos, SP, Brazil) in a 1:3 weight ratio to simulate the dilution that occurs in human saliva.^20^ Ten new soft-bristle toothbrushes (Select, Guarulhos, SP, Brazil) were dedicated to each group. The brushes were replaced for each experimental group to prevent cross-contamination between the toothpastes.

For controlled abrasion by simulated brushing, samples were mounted individually on acrylic bases placed in an automatic brushing machine (MSEt, 1500 W, Marcelo Nucci ME, São Carlos, SP, Brazil). The equipment was set to simulate brushing at a temperature of 37 °C, a speed of 4.5 Hz (full back-and-forth movement), and a load of 200 g-force (gf) [12].

The brushing process comprised a total of 5,000 reciprocal movements, which lasted for approximately 18 min 27 s. The brushing procedure was divided into two phases, each comprising 2,500 reciprocal movements. Between each brushing step, the samples were individually immersed in 3 mL of diluted toothpaste from their respective groups for 1 min 45 s. This ensured that the samples were fully immersed in the suspensions.^20^ All the specimens were abundantly rinsed with deionized water and air-dried to ensure the complete removal of any residual particles before μ-EDXRF and contact angle measurements were performed.

### 2.5 Postbrushing Analysis

The μ-EDXRF analysis following simulated brushing (second analysis) (n = 50) was conducted using the same parameters applied in the initial analysis. After the data acquiring process was complete, the data were processed using Shimadzu micro EDX MP ver. 1.03 (Shimadzu). The contact angle (wettability) of the composite resin surfaces after brushing (second analysis, n = 50) was determined using the same parameters as in the initial analysis.

### 2.6 Scanning Electron Microscopy (SEM)

To analyze the surface morphology of the resin, a representative sample from each group was selected and metallized with a layer of gold. Each sample was examined by SEM (EVO-MA10, Carl ZeissVR STM, Oberkochen, BW, Germany) with an acceleration voltage of 20 kV. The SEM images were captured at magnification 5.000× to verify possible surface modifications resulting from the treatments. The term “nano-hybrid” refers strictly to the commercial and structural classification of the composite resin utilized in this study (FORMA, Ultradent). SEM imaging at 5,000× magnification was intentionally selected to evaluate microtopographical surface degradation—such as organic matrix erosion and filler particle exposure—rather than to resolve individual nanoparticles. One composite sample was prepared and left unbrushed (UN) to compare with brushed samples regarding changes in surface morphology.

### 2.7 Abrasive Particle Characterization

The particulate fraction of each dentifrice was analyzed by mixing 20 g of the dentifrice with 50 mL of ultrapure water. To obtain the particle fraction, 1 mL of each mixture was centrifuged at 5,000 rpm for 5 min. The resultant solid material was collected and then dried in an incubator at 37 °C for 24 h. Half of the solid material was set aside for SEM analysis, while the other portion was used for μ-EDXRF and Fourier-transform Raman spectroscopy (FT-Raman). For the SEM analysis, the particles were placed on stubs and sputter-coated with gold. SEM characterization was conducted at a magnification of 750x and 5,000x with a voltage of 20 kV (EVO-MA10, Carl ZeissVR STM).

### 2.8 Spectroscopic Characterization of Dentifrices

To perform spectroscopic characterization, the dentifrice sample was taken directly from the toothpaste tube and placed in an aluminum holder without any prior preparation. The inorganic content of the toothpaste samples was analyzed using μ-EDXRF (model EDX 1300; Shimadzu) to determine the chemical composition of the main elements present in the selected samples. The analysis was conducted at a voltage of 50 kV. The inorganic and organic contents of the toothpaste samples were analyzed using FT-Raman spectroscopy (RFS 100/S – Bruker, Karlsruhe, Germany). The samples were excited with an air-cooled Nd:YAG laser (λ = 1064.1 nm). The power of the Nd:YAG laser incident on the sample was 100 mW. The spectral range obtained was from 100 to 4000 cm^–1^. The spectral resolution was set to 4 cm^–1^, and for each sample, one spectrum was collected with 100 scans.

### 2.9 Data Processing and Statistical Analysis

The average contact angle and μ-EDXRF data obtained from the composite resin were used for both pre- and post-treatment analyses.

The weight percentages of zirconia (Zr), titanium (Ti), barium (Ba), and silica (Si) were obtained after processing data using equipment software (microEDX MP ver. 1.03, Shimadzu, Kyoto, Japan).

The Kolmogorov–Smirnov test was used to assess whether the contact angle and μ-EDXRF data followed a normal distribution (GraphPad Prism, GraphPad Software, San Diego, CA, USA). The statistical comparisons between pre- and post-treatment μ-EDXRF data were performed using the Friedman test and Dunńs multiple comparison test because data were not normally distributed. The statistical comparisons between pre- and post-treatment of contact angle data were performed using one-way ANOVA and pairwise comparisons and Dunnett’s multiple comparison tests due to the normality of data.

For the qualitative spectral analysis, the FT-Raman spectra of dentifrices were baseline-corrected using Microcal Origin 6.0 software (Microcal Software, Northampton, MA, USA).

## 3 Results

### 3.1 μ-EDXRF Analysis

Significant differences in the oxygen content were observed exclusively in the CN group when comparing baseline resin composition to postbrushing data. The weight percentage of oxygen decreased after brushing (Figure 1a, *p* < 0.01). Conversely, the weight percentage of silicon (Si) increased in the CN group following brushing (Figure 1c, *p* < 0.001). Additionally, the weight percentages of barium (Ba) and titanium (Ti) increased after brushing in the AS (*p* < 0.05) and CN (*p* < 0.01) groups, respectively (Figure 1).

No other group exhibited significant changes in elemental composition from pre- to postbrushing. Furthermore, a comparison of postbrushing values across all groups revealed no significant intergroup differences in elemental composition (Figure 1).

### 3.2 Contact Angle Analysis (Wettability)

A comparison of the initial and final measurements revealed significant differences among the treatment groups. In the saliva group, the contact angle (CA) increased by 16% after brushing (** *p* < 0.01) (Figure 2a). By contrast, the CT and DT groups showed significant decreases in CA of 31% and 23%, respectively (*** *p* < 0.001), after brushing (Figure 2a). The CN and OZ groups did not show any statistically significant changes in CA after brushing (ns *p* > 0.05).

**Figure 2:**
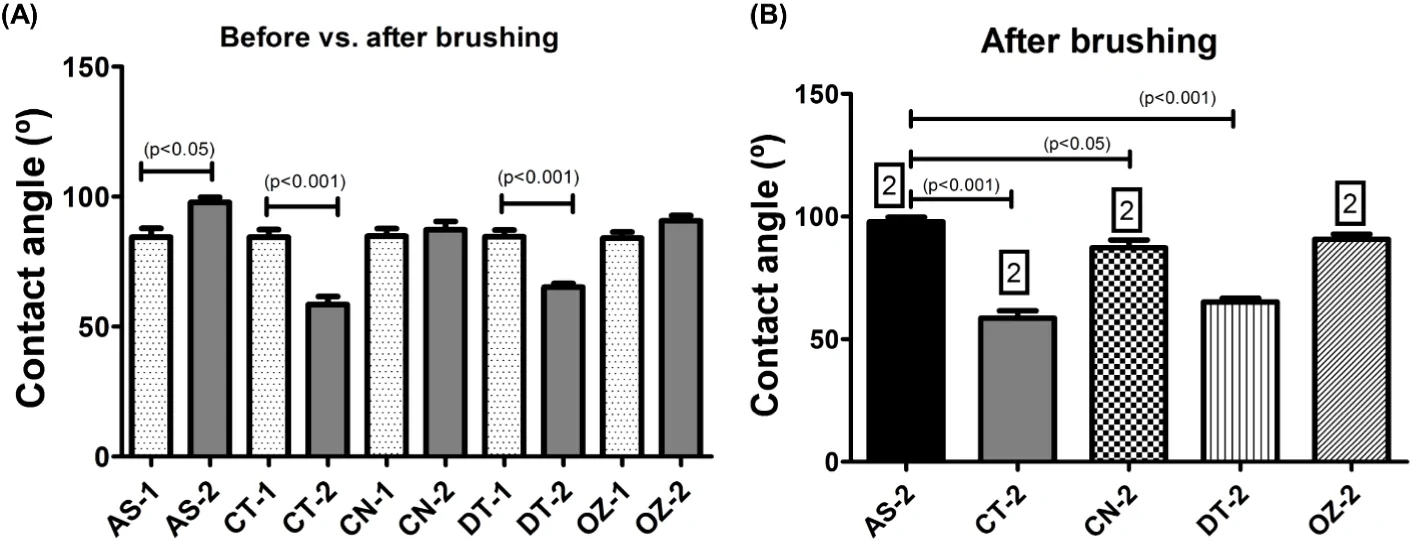
Mean and standard deviations of the contact angles (CA) before and after brushing in the experimental groups (A). The bars at the top of the figure indicate statistically significant differences between the initial and final measurements after brushing. Results from the comparative analyses of CA after brushing with comparisons between the negative and positive control groups and the experimental groups (B). The bars at the top of this figure (B) highlight statistically significant differences between the negative control group (AS) and the other groups following brushing. Equal numbers denote statistical significance between the positive control group (CT) and the other experimental groups. Legend: AS—artificial saliva, CT—Colgate Total 12 Clean Mint Control, DT—DoTERRA On Guard, CN -Colgate Natural Extracts Reinforced Defense Toothpaste, and OZ—OzoneCare Toothpaste.

Statistical comparisons of the final CA measurements after brushing revealed significant differences between the negative control group (AS) and the other groups, except for the OZ group (Figure 2b, ns *p* > 0.05). Similarly, statistical comparisons of the final CA measurements after brushing revealed significant differences between the positive control group (CT) and the other groups, except for the DT group (Figure 2b, ns *p* > 0.05).

### 3.3 SEM Analysis

Micrographs of the resin surface revealed qualitative morphological changes after the treatment. Distinct topographical differences were observed between the essential oil groups and the control groups, both negative (saliva) and positive (CT) (Figure 3). The unbrushed sample (UN, Figure 3a) exhibited a uniform surface, in contrast to the other groups. The negative control (saliva) group displayed a uniform surface in the micrographs, with no evidence of structural loss, pores, or exposure of inorganic particles. Brushstroke marks from toothbrush bristles were clearly visible (Figure 3b).

**Figure 3:**
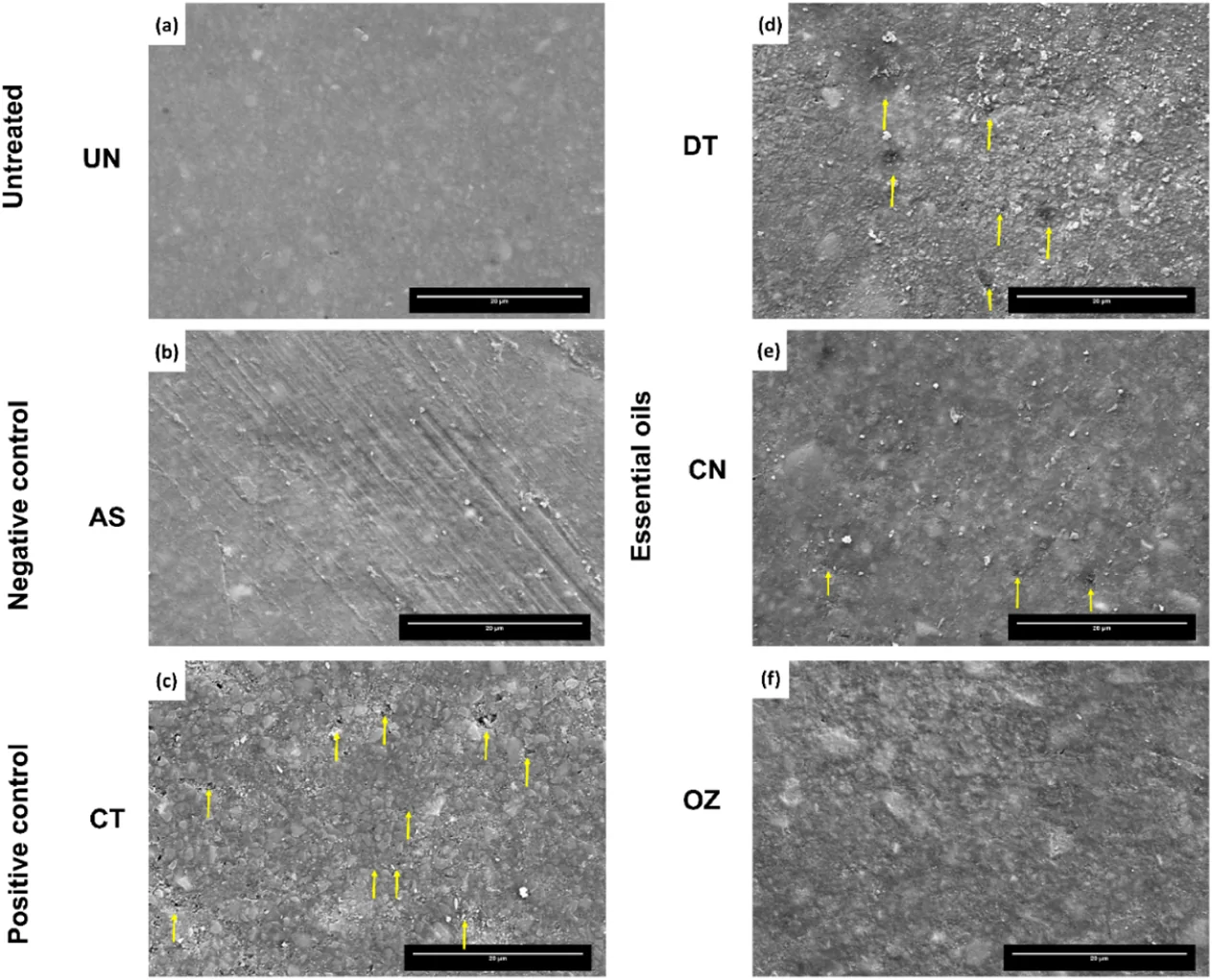
SEM micrographs (×5 000; scale bar = 20 μm) of resin surfaces: (A) unbrushed control composite resin with a smooth, homogeneous surface; (B) AS—Negative control (saliva), fine brush marks, no degradation; (C) CT—Positive control (conventional toothpaste), extensive matrix breakdown and filler exposure (yellow arrows); (D) DT—Essential-oil toothpaste, moderate surface alterations; (E) CN—Essential-oil + fluoride toothpaste, minimal topography changes; (F) OZ—Ozonated-oil toothpaste, surface closely resembles untreated resin.

By contrast, the positive control group (CT dentifrice) showed extensive degradation of the resin’s organic matrix, with exposed filler particles and areas of resin mass loss across the surface (Figure 3c, yellow arrows). The dental gel products, CN and OZ, resulted in less pronounced changes to the resin surface morphology (Figure 3d and f). The resin brushed with the DT dentifrice showed an intermediate level of alteration to its surface morphology (Figure 3e). Meanwhile, the resin treated with the OZ gel product exhibited characteristics similar to the nonbrushed sample, with minimal degradation of the resin matrix and limited exposure of filler particles (Figure 3f).

### 3.4 Abrasive Particle Characterization

SEM analysis of the isolated abrasives (Figure 4) revealed that the CT particles were larger and had more irregular shapes. By contrast, the DT, CN, and OZ particles (all of which are essential-oil-based) were smaller and uniform. Furthermore, CN and OZ (gel formulations) exhibited the most homogeneous particle shapes.

**Figure 4:**
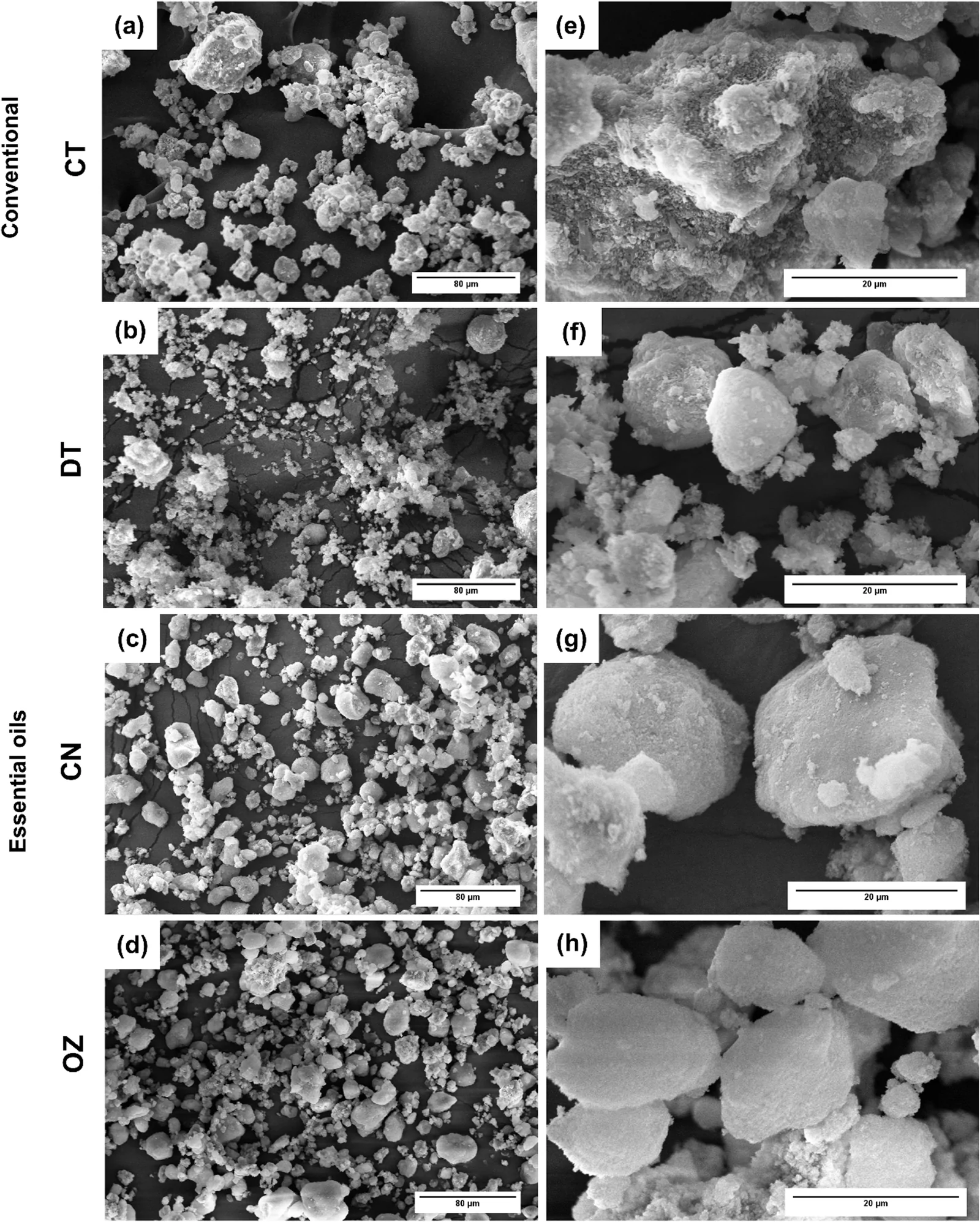
Micrographs of the abrasive grains isolated from the toothpaste of groups: CT—Colgate Total 12 Clean Mint Control (A, E), DT—DoTERRA On Guard (B, F), CN -Colgate Natural Extracts Reinforced Defense Toothpaste (C, G), and OZ—OzoneCare Toothpaste (D, H). The samples from the CT group displayed larger and more irregular particles compared to the other groups. The magnification bars indicate scales of 80 μm (750×) and 20 μm (5000×).

### 3.5 Spectroscopic Characterization of Dentifrices

The μ-EDXRF spectra of the dentifrices (Figure 5) reveal the composition of the primary inorganic particles in the powders from each sample group. The analysis indicates that silica (Si) is the main inorganic component in most dentifrices, except for the DT group, which mainly contains calcium (Ca) as its primary inorganic component. Additionally, the CT group spectrum also shows the presence of zinc (Zn), titanium (Ti), and sulfur (S) (Figure 5).

**Figure 5:**
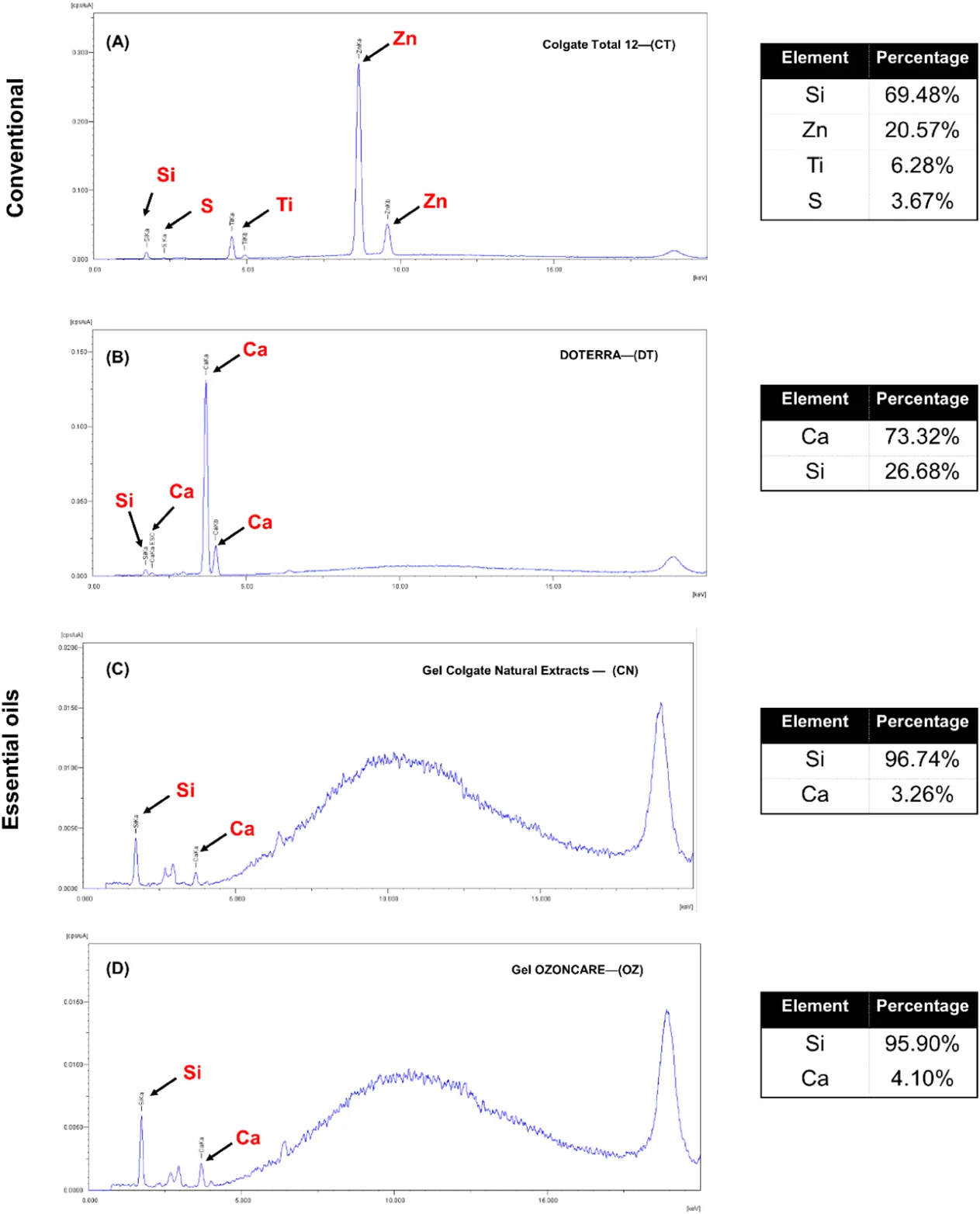
μ-EDXRF spectra with the main inorganic components from the toothpaste of groups: CT—Colgate Total 12 Clean Mint Control (A), DT—DoTERRA On Guard (B), CN -Colgate Natural Extracts Reinforced Defense Toothpaste (C), and OZ—OzoneCare Toothpaste (D). The unidentified peaks in the spectra correspond to the Rhodium (Rh) tube of the equipment or the fluorescence emitted by organic compounds present in the materials. The results of the analysis of the inorganic components are presented in the respective tables to the right of the figures.

The FT-Raman spectra of the investigated dentifrices and their respective vibrational assignments are summarized in Table 2 and showed in Figures 6 and 7. In the low-frequency inorganic region (100–1200 cm^–1^), under the applied measurement conditions, the CT dentifrice exclusively exhibited intense vibrational bands assigned to TiO_2_ at 142, 396, 508, 637, and 851 cm^–1^. A distinct peak attributed to glassy silicates appeared at 775 cm^–1^ in the DT group, while symmetric Si–O–Si stretching vibrations at 481 cm^–1^ were identified in DT and OZ. Calcium carbonate bands were observed at 871 cm^–1^ for CN and at 1085 cm^–1^ for DT. Peaks associated with C–O bond stretching in SiO_2_ and glycerin were recorded between 1030 and 1150 cm^–1^ in CT, CN, and OZ (Figure 6).

**Table 2:** FT-Raman Spectra of Dentifrices Description

Raman peak/range (cm^–1^)	Component attribution/Reference
142	TiO_2_ -^27^
396	
481	Symmetric stretching or bending vibrations of silicon–oxygen-Silicon (Si–O–Si) -^29^
508	
637	TiO_2_ -^27^
775	Glassy silicates -^29^
851	TiO_2_ -^27^
851	Essential oils, ring stretching vibrations or C–H deformations -^30^
871	Calcium carbonate -^26^
1030–1150	SiO_2_ -^32^ Glycerin, stretching vibrations of the C–O bonds -^31^
1085	Calcium carbonate -^28^
1461, 1465	CH_3_ bonds -^26^
2892	CH_2_ bonds in glycerin -^26^
2924	Polyethylene glycol 12 (PEG-12) -^26^
2940	CH_2_ bonds in glycerin -^26^
3000–3500	OH stretching vibrations of H_2_O -^26^

**Figure 6:**
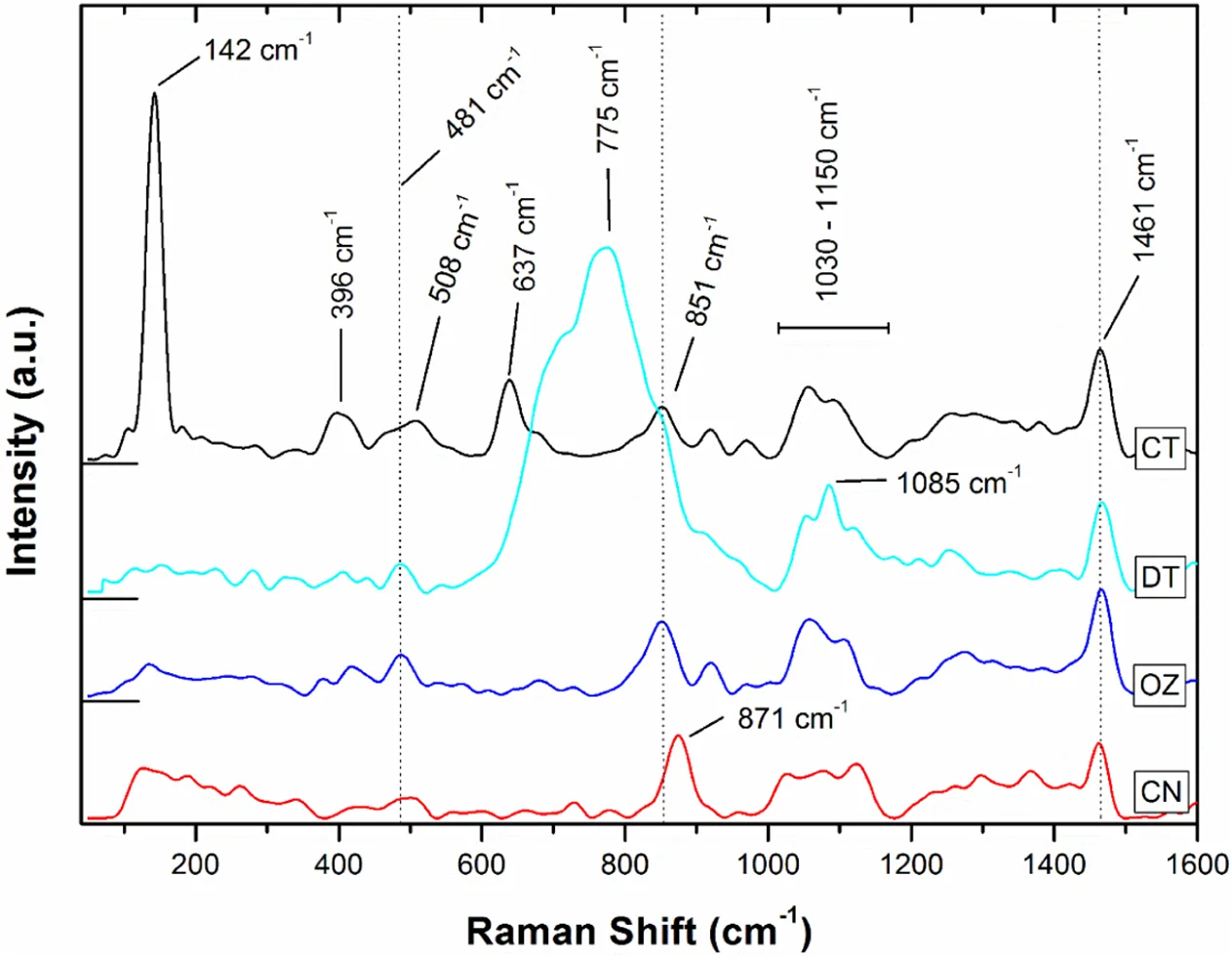
FT-Raman stacked spectra of the evaluated dentifrices at low-frequency region (100–1600 cm^–1^) highlighting the distinct inorganic markers, notably the intense TiO2 bands in the CT group (142, 396, 508, 637 cm^–1^) and glassy silicates in the DT group (775 cm^–1^). Spectra were shifted for clarity. Legend: CT—Colgate Total 12 Clean Mint Control, DT—DoTERRA On Guard, CN -Colgate Natural Extracts Reinforced Defense Toothpaste, and OZ—OzoneCare Toothpaste.

**Figure 7:**
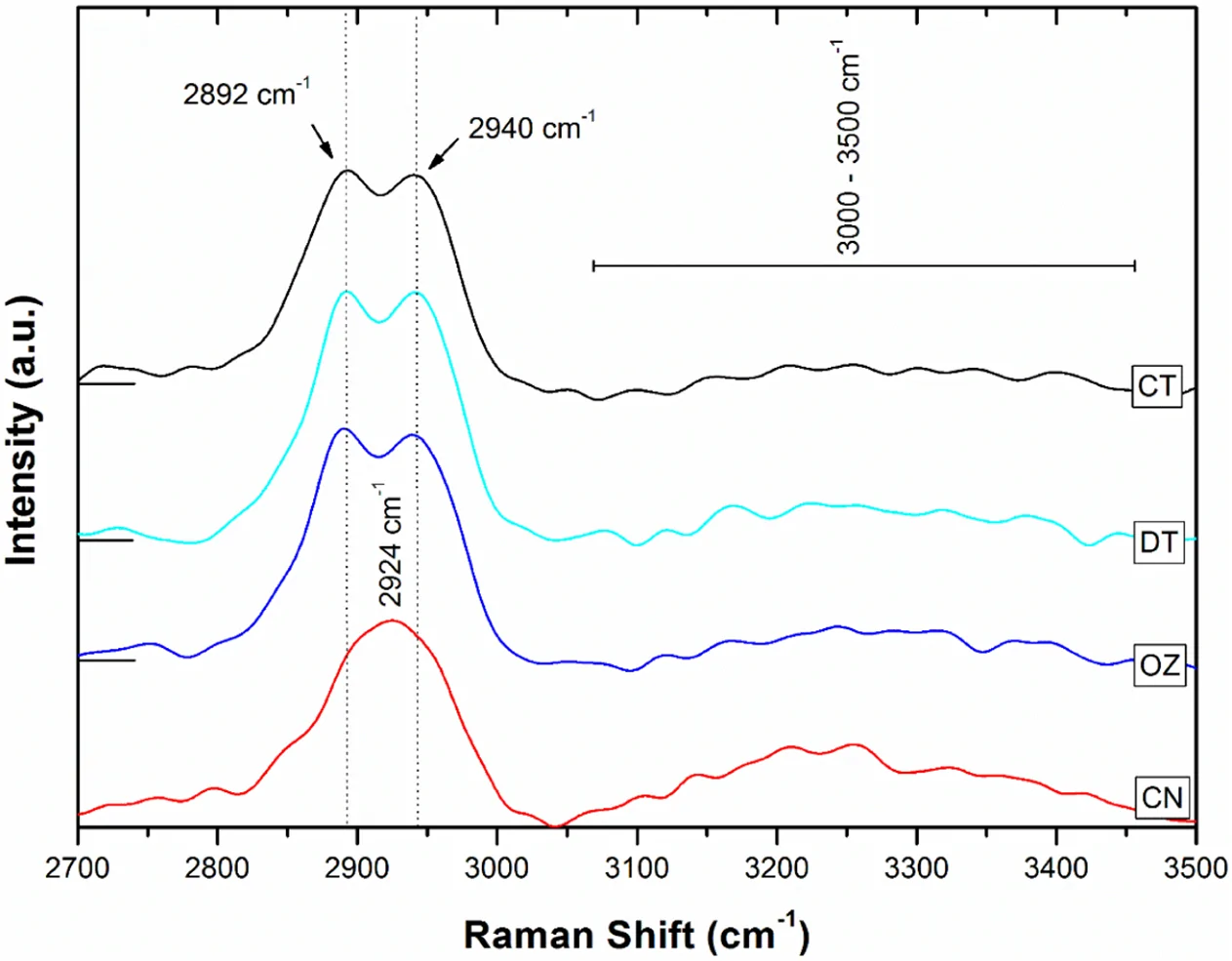
FT-Raman stacked spectra of the evaluated dentifrices at the high-frequency region (2700–3500 cm^–1^) differentiating the organic humectant vehicles, with the CN formulation demonstrating a unique PEG-12 signature (2924 cm^–1^) compared to glycerin-based matrices of CT, DT, and OZ (2892 and 2940 cm^–1^). Spectra were shifted for clarity. Legend: CT—Colgate Total 12 Clean Mint Control, DT—DoTERRA On Guard, CN -Colgate Natural Extracts Reinforced Defense Toothpaste, and OZ—OzoneCare Toothpaste.

In the organic spectral region, C–H deformations and ring stretching modes associated with essential oils were detected at 851 cm^–1^ in the OZ group (Figure 6). Bending modes corresponding to CH_3_ groups were identified at 1461–1465 cm^–1^ across all dentifrices. In the high-frequency C–H region (Figure 7), characteristic peaks at 2892 and 2940 cm^–1^ (CH_2_ bonds in glycerin) were present in CT, DT, and OZ, whereas a distinct peak at 2924 cm^–1^ (assigned to polyethylene glycol-12 (PEG-12)) was recorded exclusively for CN. Finally, a broad absorption band between 3000 and 3500 cm^–1^, corresponding to O–H stretching vibrations of water, was identified in all dentifrice spectra (Figure 7).

To visually synthesize the spectral distribution of the chemical components across the experimental groups, a binary heat map was constructed based on the FT-Raman analysis (Figure 8). The map clearly clusters the formulations into distinct inorganic and organic profiles. In the inorganic region, the CT dentifrice exhibits a dense spectral footprint corresponding exclusively to TiO_2_, whereas DT and CN show distinct markers for glassy silicates and calcium carbonates. Conversely, the OZ formulation lacks these heavy crystalline abrasive markers, presenting only Si–O–Si stretching vibrations and SiO_2_/glycerin C–O bonds. In the organic region, the humectant vehicles are distinctly mapped: the glycerin CH_2_ bonds are prominently present in CT, DT, and OZ, while the CN formulation is uniquely characterized by the PEG-12 marker. Baseline organic markers for CH_3_ bonds and H_2_O were uniformly identified across all of the groups.

**Figure 8:**
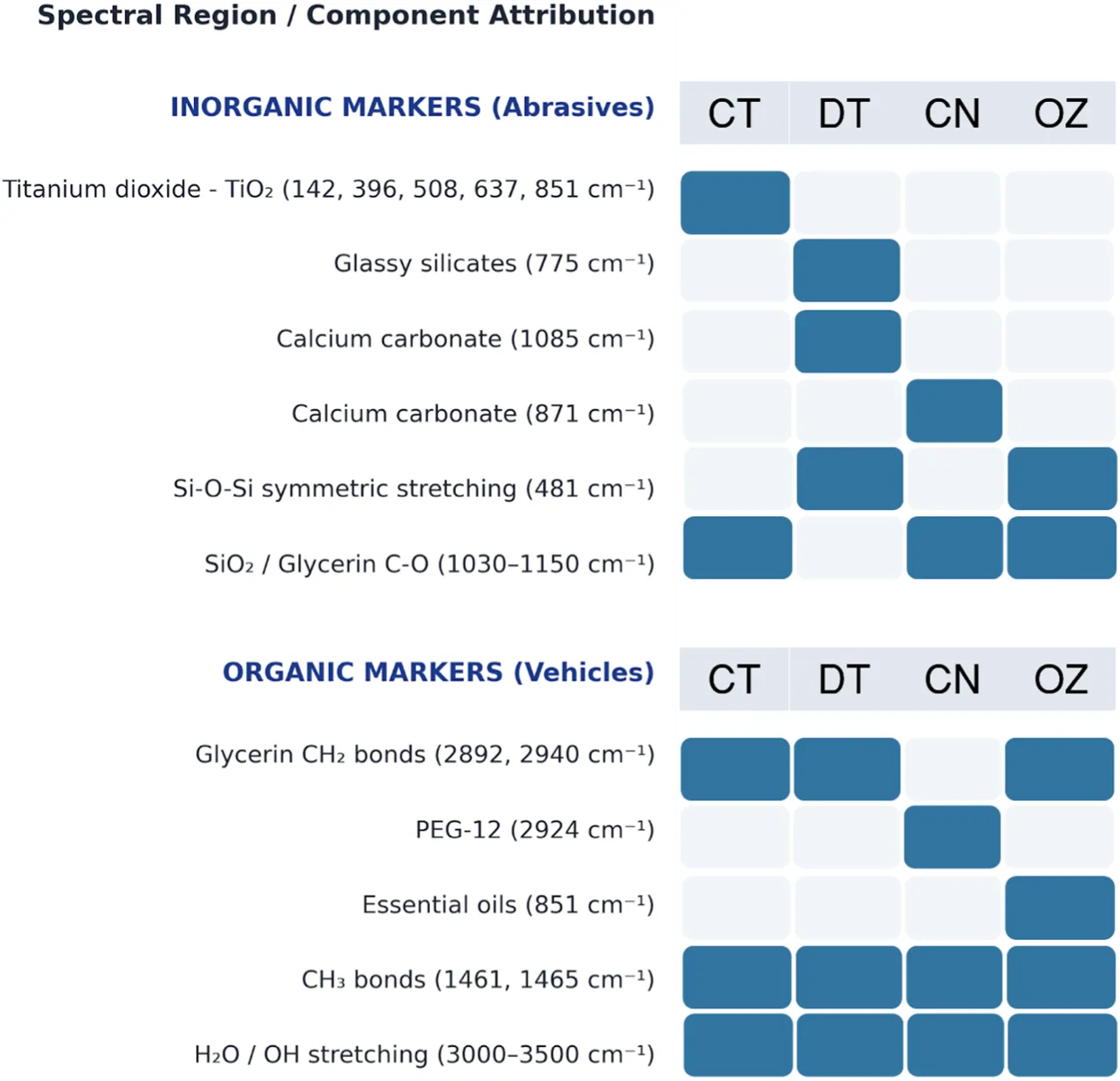
Spectral heat map illustrating the distribution of inorganic and organic markers across the evaluated dentifrices. Blue blocks indicate the presence of specific FT-Raman vibrational modes, while gray blocks indicate their absence. The map visually clusters the distinct abrasive profiles, contrasting the heavy crystalline inorganic footprint of CT and DT with the predominantly organic and humectant-based matrix signature of the OZ gel formulation. Legend: CT—Colgate Total 12 Clean Mint Control, DT—DoTERRA On Guard, CN -Colgate Natural Extracts Reinforced Defense Toothpaste, and OZ—OzoneCare Toothpaste.

**Figure 9:**
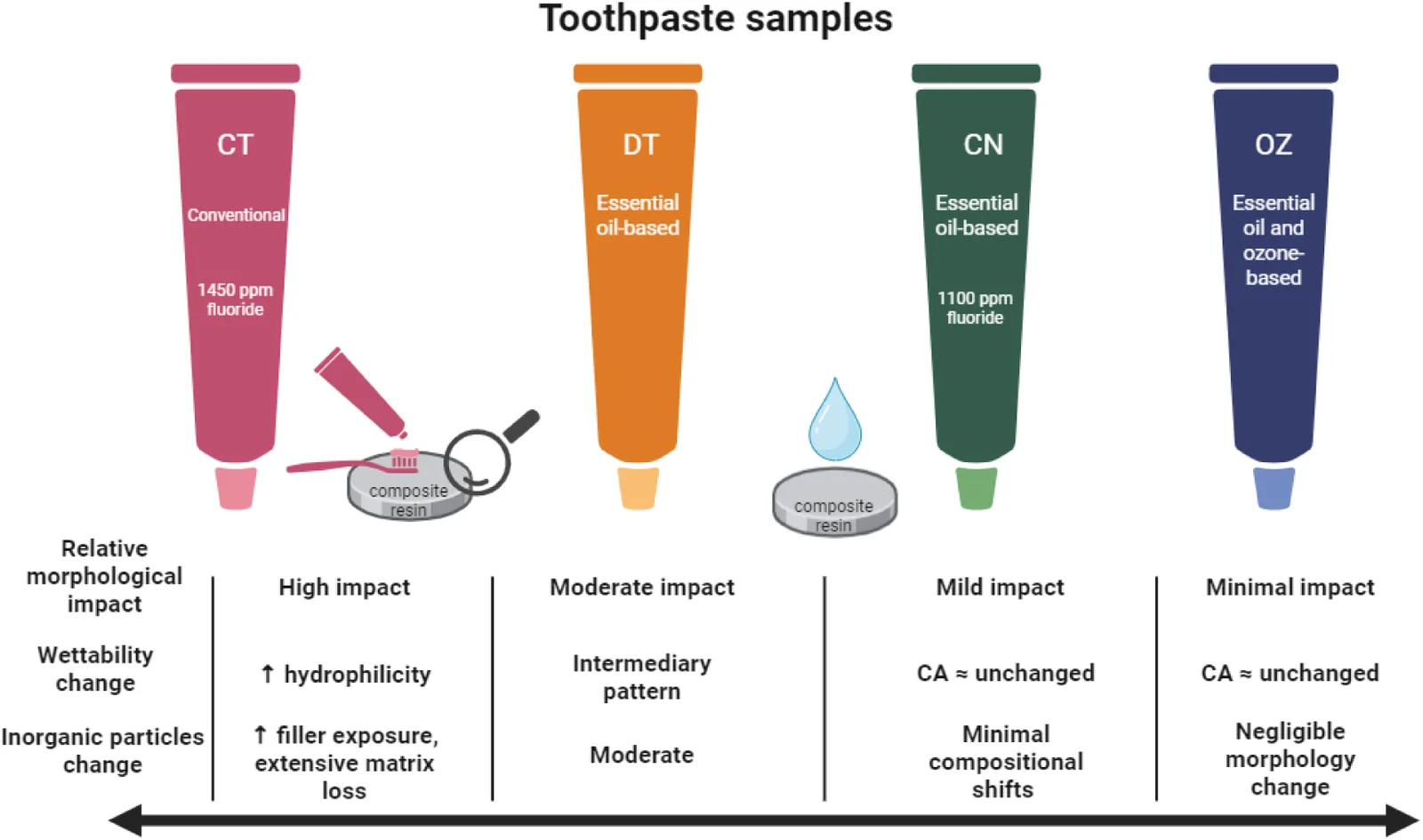
Schematic illustration of the features and results found on composite resin after brushing in each group: CT—Colgate Total 12 Clean Mint Control, DT—DoTERRA On Guard, CN -Colgate Natural Extracts Reinforced Defense Toothpaste, and OZ—OzoneCare Toothpaste.

## 4 Discussion

This study evaluated the effect of simulated toothbrushing on the chemical, physical, and morphological features of nanoparticle-reinforced composite resin. The data collected in this study support the formulated initial hypothesis. The consistency and composition of toothpaste had significant differences, which affected the properties of the materials tested.

Research on resin degradation caused by oral hygiene products is important because composite resin is a type of dental material prone to sorption and solubility. These two processes lead to other chemical and physical changes that pose biological risks and can damage the resin’s structure and function.^1,11^

This study combined μ-EDXRF, contact-angle goniometry, SEM, and FT-Raman spectroscopy to comprehensively assess how novel essential-oil and ozonated-oil dentifrices influence a zirconia-reinforced nanohybrid composite.

The analysis was structured sequentially into baseline characterization, brushing simulation, and post-treatment evaluation—to elucidate both the mechanical and the chemical effects of these formulations on the composite resin.

Baseline μ-EDXRF and contact-angle measurements confirmed the homogeneity of all 50 resin specimens before brushing, validating our sample preparation protocol. Uniform elemental distributions (O, Si, Ba, Ti) and consistent wettability (CA ∼ 75°) were observed for the glass hybrid composite.

The samples subjected to brushing with artificial saliva were used as a negative control. After 5,000 simulated brushing cycles, only the artificial saliva (AS) and fluoride-reinforced essential oil (CN) groups showed significant changes in their μ-EDXRF elemental profiles. In the CN group, there was a decrease in oxygen (O). The AS and CN groups showed an increase in silicon (Si), barium (Ba), and titanium (Ti).

These changes are due to modifications in the organic portion of the resin, which is more susceptible to chemical degradation than the inorganic material. The μ-EDXRF technique enables indirect evaluation of modifications to the inorganic matrix by analyzing changes in the proportion of inorganic elements. When shifts in these inorganic element proportions are observed, it reflects changes that initially occurred in the organic content, as the resin matrix’s organic portion is used as a stoichiometric balance during equipment calibration.^1^

In the AS group, these changes were attributed to the process of water sorption, plasticization, and hydrolysis of the composite resin matrix, resulting from prolonged exposure to the aqueous medium. Thus, the artificial saliva components and water sorption by the resin matrix probably resulted in plasticization, softening, and hydrolysis of the material, thus promoting greater matrix dissolution and exposure of the inorganic fillers.^5,12^

These changes compare to what clinicians observe in a typical restoration’s lifespan, where gradual wear and degradation of the resin can lead to increased exposure of fillers and potential failure of the restoration. Framing these findings with the lifespan of a typical restoration helps professionals understand the implications of the degradation rates observed in the present study. Another possible influence in the AS group was the direct contact of brushing bristles to the composite resin surface, without the presence of the dentifrice between them. In addition to the dentifrice abrasivity, the bristles of the dental brush also cause degradation of the composite.^9^

Although softer bristles lead to a lower level of degradation of the composite surface, time and brushing force contribute to altering the restorative material surface.^10^

Wettability data parallel these findings. The simulated brushing with artificial saliva and without any dentifrice resulted in a significant increase in the contact angle (CA) by 16% (*p* < 0.01), which is probably related to the process of water sorption, plasticization, and hydrolysis of the composite organic matrix.

For the CN group, the surfactants and essential oil solvents in the gel vehicle similarly softened the resin domains, allowing the fillers to enrich at the surface. However, these alterations were likely initial and do not indicate significant changes in wettability (Figure 2).

In contrast, the conventional (CT), nonfluoride essential oil (DT), and ozonated oil (OZ) groups showed no compositional changes. Despite this, the contact angle and surface wettability were significantly influenced by brushing with CT and DT. Only the OZ group had no notable changes in composition or wettability. This implies that these formulations exert different effects on the features of composite resin, with higher (CN, CT, and DT) and lower (OZ) chemical aggressiveness.

The abrasiveness of the dentifrices influenced the surfaces of the composite materials under study. Increased dentifrice abrasiveness resulted in greater material wear and, consequently, higher surface roughness.^6^ SEM images visually corroborate the quantitative findings: CT produced the most significant surface damage, DT caused intermediate damage, and CN/OZ caused the least damage.

The organic matrix of a composite resin is worn by brushing, which exposes the underlying inorganic particles,^8^ as seen in the qualitative analysis in this study. Previous studies indicated that dentifrices containing natural substances, such as essential oils, influence both enamel and dentin features.^13,14^ Nevertheless, a notable gap exists regarding these products—especially ozonated oil formulations—and their impact on composite resin restorations. The available studies are scarce or absent, limiting the applicability of enamel findings to restorative materials. This study addresses that gap by directly investigating the effects of alternative dentifrices on zirconia-reinforced composites, delivering crucial preliminary evidence to inform clinical guidance and stimulate further longitudinal and translational clinical research.

In the present study, the gel with ozonated oil (OZ) showed minimal compositional and topographical changes compared to those of conventional formulations and some formulations based on essential oils, suggesting a conservative interaction with the restorative surface.

The μ-EDXRF data showed no significant postbrushing shifts in O, Si, Ba, or Ti for the OZ group, indicating preservation of the inorganic fraction of the composite resin and limited removal of the organic matrix. This result is corroborated by SEM micrographs, where OZ group surface features closely resemble unbrushed control and show only striations without matrix loss or filler exposure.

Formulation factors likely explain the low-impact profile found in the OZ group: gel vehicles improve abrasive suspension and lubrication during brushing, reducing direct particle-to-surface impacts, while fine, homogeneous abrasive distributes contact forces more evenly and minimize localized plowing. The particle morphology and formulation rheology are established as determinants of abrasive behavior in oral formulations.^21,22^

Ozonated oils also form transient surface films or alter interfacial energy, which is hypothesized to lower abrasive stress on the resin matrix by providing a protective layer at the composite interface. This sequence, where modified interfacial properties decrease binder solubilization and plucking during brushing, underpins the proposed protective mechanism. However, direct molecular-level evidence in dental substrates remains limited, necessitating targeted surface chemistry studies, such as X-ray photoelectron spectroscopy (XPS), to confirm these proposed protective mechanisms.^21^

For patients invested in esthetic restorations, using the OZ formulation is an option to help maintain both composite restorations and enamel surfaces by minimizing damage caused by brushing. Clinically, the minimal chemical and morphological changes observed after brushing in the OZ group suggest that this formulation has the potential to better preserve these surfaces in patients with extensive esthetic work, since limiting organic matrix loss and filler exposure reduces the increases in surface roughness that favor biofilm retention and staining.^23^

A conservative dentifrice profile requires balancing against cleaning efficacy: low-abrasivity pastes protect surfaces but often underperform in stain removal, whereas highly abrasive formulations remove stains at the cost of surface wear.^22^ The present in vitro study indicates conservative material preservation in the OZ group. Despite this, clinical trials are necessary to verify that cleaning and plaque control outcomes remain acceptable in vivo.

Comparison with higher-abrasivity formulations in this and other studies show concordant patterns. The conventional dentifrices containing large, irregular abrasives or TiO_2_ tend to produce pronounced matrix loss and filler exposure.

Recent advances in dental materials highlight the exceptional mechanical properties, chemical stability, and wear resistance of zirconia, making it a highly suitable structural component for contemporary dentistry. Furthermore, the incorporation of titanium dioxide (TiO_2_) into zirconia-based ceramics has been shown to synergistically enhance the structural integrity,^24^ wear resistance, and antimicrobial efficacy.^24,25^ However, our findings reveal a different biomechanical dynamic when these robust materials interact frictionally on the restorative surface. While TiO_2_ provides excellent wear resistance and chemical stability as an integrated structural dopant, its presence as a hard, irregular abrasive particle in conventional toothpastes (such as the CT group) acts aggressively against the softer organic matrix of the zirconia-reinforced composites. The frictional interaction explains the pronounced matrix loss and subsequent exposure of the underlying zirconia-silica fillers observed in our SEM analysis (Figure 3c).

In contrast, some charcoal-containing products with large, angular carbon particles induce deep grooves and rapid roughness increases. Particle size, shape, and composition therefore strongly determine damage patterns.^4^

Literature reports advantages in using natural or ozone-containing dentifrices, which resulted in intermediate-to-low roughness and limited color change compared with highly abrasive charcoal pastes.^13^ Campolina et al.^13^ found that some natural/ozone formulations induced smaller roughness and color shifts than high-abrasivity charcoal products, supporting formulation-based guidance for clinical use.

This study provided detailed chemical insights into the composition of dentifrices by employing μ-EDXRF to assess inorganic components (Figure 5) and FT-Raman spectroscopy to characterize both organic vehicles and inorganic filler matrices (Table 2, Figures 6–8). These spectroscopic techniques yielded composition data that are directly relevant to the observed results and potential clinical applications. μ-EDXRF and contact angle analyses demonstrated that the composition and wettability of the zirconia composite material were significantly affected by the CN, CT, and DT products. Spectroscopic characterization of the dentifrices (Figures 5 and 6) identified titanium oxide, tetrasodium phosphate, and calcium carbonate, which explain the substantial changes observed in the composite resin following brushing in CN, CT, and DT groups.

It is important to note the specific analytical scope of the techniques employed in this study. While μ-EDXRF provides highly precise elemental mapping (e.g., detecting variations in elemental Titanium), it identifies isolated elements rather than specific molecular compounds. To complement this elemental profile, FT-Raman spectroscopy was utilized for molecular characterization.

Cross-referencing the spectroscopic characterization (Table 2, Figures 6–8) with the reported product compositions (Table 1) successfully elucidated the structural variations among formulations. FT-Raman spectroscopy confirmed the vehicle rheology and humectant bases: the presence of glycerin in CT, DT, and OZ was verified by distinct CH_2_ stretching modes (2892 and 2940 cm^–1^),^26^ whereas CN exhibited a unique peak at 2924 cm^–1^ corresponding to PEG-12,^26^ corroborating with the manufacturer’s specifications (Figure 7).

Crucially, FT-Raman spectroscopy identified the specific crystalline features of the abrasive agents responsible for the observed physical degradation of the composite surface. While hydrated silica serves as the baseline abrasive across all formulations, the CT spectrum was heavily dominated by intense titanium dioxide (TiO_2_) crystalline bands (142, 396, 508, 637, and 851 cm^–1^).^27^

Interestingly, although TiO_2_ is listed in the formulation for CN, no detectable Raman band at 142 cm^–1^ was observed under the applied measurement conditions, likely due to a lower concentration below the equipment detection limit or signal masking by the fluorescent PEG-12/sorbitol gel vehicle. Consequently, the severe morphological alterations observed in the CT group (Figure 3) can be directly correlated to the detectable presence of these large, structural TiO_2_ abrasive particles, which were not functionally evident in the CN spectra.

The presence of large, crystalline TiO_2_ particles acting as hard abrasives in CT, alongside calcium carbonate (1085 cm^–1^)^28^ and glassy silicates (775 cm^–1^)^29^ in DT, provides a clear chemical rationale for the severe topographic modification, organic matrix erosion, and filler exposure observed in SEM analysis after brushing (Figure 3). Conversely, the OZ formulation displayed a milder spectroscopic footprint characterized by Si–O–Si network vibrations (481 cm^–1^),^29^ essential oil modes (851 cm^–1^),^30^ and glycerin (1030–1150 cm^–1^)^31^ (Figure 6), supporting its conservative interaction with the restorative matrix.

The binary heat map derived from the FT-Raman spectra (Figure 8) provides a compelling visual correlation between the chemical formulations and the topographical alterations observed on the composite resin. By clustering the spectral data, it becomes evident that formulations producing the highest morphological impact (CT and DT) are characterized by a dense footprint of hard inorganic markers—specifically the intense TiO_2_ block in CT and the silicate/carbonate bands in DT. These crystalline abrasives act aggressively against the softer resin matrix during brushing. In contrast, the OZ formulations show a minimal inorganic presence, lacking aggressive crystalline markers while still emphasizing its organic components like essential oils and glycerin. This spectral clustering supports the morphological results: the absence of heavy abrasive markers in the OZ heat map indicates the composite matrix remains intact. Meanwhile, the dense inorganic clusters in CT and DT align with the matrix erosion and filler exposure seen in the SEM analysis (Figure 3).

Gel dentifrices that utilize silica as the abrasive agent typically exhibit lower abrasiveness compared to other dentifrices.^10^ When silica is combined with other abrasives, such as calcium carbonate, sodium pyrophosphate, titanium oxide, and sodium phosphate, the resulting dentifrice is classified as highly abrasive. In contrast, silica in fine, regularly shaped particles retains its characteristic as a low-abrasive mineral. The incorporation of thick, irregular particles increases the abrasiveness of the dentifrice. Therefore, the formulation or type of abrasive alone does not adequately characterize the abrasiveness of a dentifrice on composite materials.^10^

The limitations of the current in vitro study include the inability to fully reproduce oral conditions, such as salivary pellicle formation, pH fluctuations, dietary staining, and patient brushing variability. These factors modulate dentifrice performance and attenuate or amplify the effects observed here, underscoring the need for longitudinal clinical trials and complementary assays (RDA/REA, profilometry, and biofilm formation tests) to comprehensively evaluate dentifrices containing essential oils and ozonated oils and comparable products.^22^

In summary, the ozonated-oil gel dentifrice demonstrated a low-impact profile on a zirconia-reinforced nanohybrid composite, preserving inorganic composition and surface morphology with minimal changes. The results of the present study support considering gel-based, low-abrasivity formulations, such as OZ product for patients with multiple restorations, provided that future clinical work confirms adequate hygiene and stain-control performance.

Different results were found in this study according to the type of dentifrice tested. Figure 9 synthesizes the multimodal results obtained. The conventional dentifrice (CT) and the essential oil paste with an abrasive profile (DT) produced the greatest surface damage, marked matrix loss, and filler exposure (high morphological impact); the fluoride-containing essential oil dentifrice (CN) generated measurable compositional shifts (moderate impact) without severe topographic modifications; the ozonated oil gel (OZ) preserved inorganic composition and surface morphology without significant filler exposure (minimal impact). Based on these findings, it is recommended that clinicians advise patients with multiple composite restorations to incorporate gel-based formulations that minimize surface degradation, such as OZ for routine use, or CN when fluoride addition is necessary, into their daily oral hygiene regimen. Higher-abrasivity products are limited to patients requiring occasional short-term stain removal. These patterns, derived from μ-EDXRF, SEM, and contact angle data, highlight formulation aspects like vehicle rheology and abrasive particle morphology as primary drivers of material response.

## 5 Conclusions

Within the limitations of this current study, it was concluded that

Brushing with the tested dentifrices produced measurable changes in the inorganic composition of the zirconia-reinforced nanohybrid composite. Specifically, the CN group showed a significant decrease in oxygen content, and the AS and CN groups showed increases in silicon, barium, and titanium.
Brushing with CT and DT produced significant reductions in the contact angle, whereas CN and OZ did not produce significant changes in wettability, and AS produced an increase in the contact angle.
SEM analysis showed extensive organic matrix erosion and filler exposure after brushing with CT, moderate surface alteration with DT, and minimal topographic changes with CN and OZ. SEM analysis revealed an extensive morphological impact and increased filler exposure in samples treated with CT, moderate degradation with DT, and minimal morphological impact with CN and OZ.
Notably, the OZ gel preserved both the elemental composition and surface morphology of the composite.
